# Editorial: Potential pathophysiological mechanisms underlying the comorbidity of depression and macrovascular diseases

**DOI:** 10.3389/fpsyt.2023.1349818

**Published:** 2024-01-05

**Authors:** Jingen Li, Kuibao Li, Joshua Bock, Haibin Zhao, Xiaodong Sun, Sen Li

**Affiliations:** ^1^Department of Cardiovascular Medicine, Dongzhimen Hospital, Beijing University of Chinese Medicine, Beijing, China; ^2^Heart Center and Beijing Key Laboratory of Hypertension, Beijing Chaoyang Hospital, Capital Medical University, Beijing, China; ^3^Department of Cardiovascular Medicine, Mayo Clinic, Rochester, MN, United States; ^4^The DongFang Hospital of Beijing University of Chinese Medicine, Beijing, China; ^5^Department of Endocrinology and Metabolism, Affiliated Hospital of Weifang Medical University, Weifang, China; ^6^School of Life Sciences, Beijing University of Chinese Medicine, Beijing, China

**Keywords:** depression, macrovascular diseases, comorbidity, risk factors, pathophysiological mechanisms

Depression, a prevalent mental disorder impacting more than 300 million people globally, is the primary contributor to disability. Macrovascular disease is a health condition that impacts the major blood vessels in the body, including the aorta, coronary arteries in the heart, and arteries in the brain and limbs. The increased prevalence of macrovascular diseases, including coronary, cerebrovascular, and peripheral vascular disease, among older individuals has substantially elevated global healthcare expenditure. There is a well-established connection between depression and specific types of macrovascular diseases. One example is the established reciprocal causation between depression and coronary disease, indicating a bidirectional amplification of risk for these conditions. Nevertheless, the mechanisms underlying the interaction between depression and macrovascular disease remain not entirely comprehended.

This Research Topic focuses on exploring the interaction mechanisms between depression and macrovascular disease, aiming to clarify their combined mechanisms and identify the underlying factors that impact this interaction. The Research Topic comprised two review articles and three original research articles, covering a broad spectrum of studies ranging from fundamental research to clinical investigations. These articles provide valuable insights to improve our comprehension of the common pathophysiology shared between depression and macrovascular disease.

Garrels et al. and Zhao et al. conducted extensive reviews exploring the connections between depression and cardiovascular conditions. Garrels et al.'s review focused on depression following myocardial infarction (MI) and its associations with various factors, including dysregulation of the autonomic nervous system, hypothalamic-pituitary-adrenal (HPA) axis, inflammatory cytokines, coagulation system, platelet aggregation, and environmental factors. Moreover, the review emphasizes that depression may serve as an adverse event for drugs used in the treatment of MI, such as beta-blockers, statins, and antiplatelet agents. Zhao et al.'s review emphasized shared pathophysiological mechanisms between macrovascular disease and depression, including neuroendocrine factors (such as HPA axis dysfunction), immune-inflammatory responses (involving proinflammatory cytokines), and platelet dysfunction (characterized by hyperactivation and aggregation). Furthermore, both reviews underscored the impact of pathological factors, such as autonomic nervous system dysfunction, on the interplay between macrovascular disease and depression.

Wang et al. employed bioinformatics analysis and experimental validation in a study to explore the pathogenesis of the co-occurrence of MI and depression at the gene level. Their results uncovered that immune inflammation could function as a common pathogenic mechanism linking MI and depression. Moreover, they pinpointed hub S-DEGs (Differentially Expressed Genes) as prospective biomarkers for the diagnosis and characterization of molecular subtypes of MI and depression.

Li et al. utilized Mendelian randomization to explore the causal connection between postpartum depression and both cerebrovascular disease and cognitive impairment. Their results demonstrated a causal link between postpartum depression and cognitive decline, while no causal relationship was identified with cerebrovascular disease.

Sleep and mood are intricately linked to mental health, and Pan et al. conducted a study to test the hypothesis that red light can influence alertness, mood, and objective sleep structure in individuals with insomnia disorders. The findings suggested that red light elevated subjective alertness, anxiety, and negative emotions in both healthy subjects and individuals with insomnia disorder. These effects can impact sleep either directly or indirectly through the mediating effect of negative emotions.

The Research Topic highlights the importance of understanding potential pathophysiological mechanisms underlying the comorbidity of depression and macrovascular diseases. These findings offer valuable insights for clinical application and disease prevention.

## Author contributions

JL: Writing—original draft, Writing—review & editing. KL: Writing—review & editing. JB: Writing—review & editing. HZ: Writing—review & editing. XS: Writing—review & editing. SL: Writing—original draft, Writing—review & editing.

